# Exploration of the effects of a *degS* mutant on the growth of *Vibrio cholerae* and the global regulatory function of *degS* by RNA sequencing

**DOI:** 10.7717/peerj.7959

**Published:** 2019-10-23

**Authors:** Jian Huang, Yuxi Chen, Jie Chen, Changjin Liu, Tao Zhang, Shilu Luo, Meirong Huang, Xun Min

**Affiliations:** 1 Department of Laboratory Medicine, Affiliated Hospital of Zunyi Medical University, Zunyi, China; 2 Department of Blood Transfusion, Affiliated Hospital of Zunyi Medical University, Zunyi, China

**Keywords:** *Vibrio cholerae*, DegS, Growth, RNA sequencing

## Abstract

**Background:**

DegS is a periplasmic serine protease that is considered to be the initiator of the σ^E^ stress response pathway, and this protein plays an important role in the regulation of the stress response in *E. coli*. However, knowledge of the biological function and global regulatory network of DegS in *Vibrio cholerae* remains limited. In this study, we aimed to characterize the molecular functions and further investigate the regulatory network of *degS* in *V. cholerae*.

**Methods:**

A deletion mutant of *degS* was constructed in the *V. cholerae* HN375 strain. Bacterial colony morphology was observed by a plate-based growth experiment, and bacterial growth ability was observed by a growth curve experiment. High-throughput RNA sequencing (RNA-Seq) technology was used to analyze the differential transcriptomic profiles between the wild-type and *degS* mutant strains. Gene ontology (GO), pathway analysis and Gene-Act-network analysis were performed to explore the main functions of the differentially expressed genes. Quantitative real-time PCR (qRT-PCR) was performed to validate the reliability and accuracy of the RNA-Seq analysis. The complementation experiments were used to test the roles of *degS* and *ropS* in the small colony *degS* mutant phenotype.

**Results:**

When *degS* was deleted, the *degS* mutant exhibited smaller colonies on various media and slower growth than the wild-type strain. A total of 423 differentially expressed genes were identified, including 187 genes that were upregulated in the *degS* mutant compared to the wild-type strain and 236 genes that were relatively downregulated. GO categories and pathway analysis showed that many differentially expressed genes were associated with various cellular metabolic pathways and the cell cycle. Furthermore, Gene-Act network analysis showed that many differentially expressed genes were involved in cellular metabolic pathways and bacterial chemotaxis. The cAMP-CRP-RpoS signaling pathway and the LuxPQ signal transduction system were also affected by the *degS* mutant. The expression patterns of nine randomly selected differentially expressed genes were consistent between the qRT-PCR and RNA-seq results. The complementation experiments showed that the small colony *degS* mutant phenotype could be partially restored by complementation with the pBAD24-*degS* or pBAD24-*rpoS* plasmid.

**Discussion:**

These results suggest that the *degS* gene is important for normal growth of *V. cholerae*. Some of the differentially expressed genes were involved in various cellular metabolic processes and the cell cycle, which may be associated with bacterial growth. Several new *degS*-related regulatory networks were identified. In addition, our results suggested that the cAMP-CRP-RpoS signaling pathway may be involved in the small colony *degS* mutant phenotype. Overall, we believe that these transcriptomic data will serve as useful genetic resources for research on the functions of *degS* in *V. cholerae*.

## Introduction

*Vibrio cholerae* is the pathogen that causes cholera. According to statistical data from the World Health Organization, there are approximately 1.4–4.3 million cases of acute diarrheal diseases worldwide per year, including 28,000–142,000 deaths (Ali et al., 2012). At present, more than 200 serotypes of *V. cholerae* have been identified based on the different O antigenic lipopolysaccharides (Chatterjee & Chaudhuri, 2003). Only the O1 and O139 serogroups are associated with epidemic and pandemic cholera (Mutreja et al., 2011), while non-O1/non-O139 serogroups can cause sporadic local cholera outbreaks, mild and transient gastrointestinal diseases and extraintestinal infections (Aydanian et al., 2015). However, there have been increasing reports of non-O1 and non-O139 diseases in recent years, and there have even been reports of death in extreme cases (Petsaris et al., 2010; Trubiano et al., 2014; Hao et al., 2015). Knowledge regarding the pathogenic mechanism of non-O1/non-O139 *V. cholerae* remains limited. Hence, a wide range of studies on both the biological characteristics and pathogenesis of non-O1/non-O139 *V. cholerae* is important.

DegS is an important molecular stress sensor in the periplasm of bacteria that strictly controls the σ^E^-dependent stress response pathway (Zeth, 2004). In *E. coli*, many misfolded proteins are produced when the bacteria are exposed to stress. These misfolded proteins can bind to the PDZ domain of DegS, leading to the activation of DegS, which cleavage of RseA initiates proteolytic cascade processes, resulting in release of σ^E^ that ultimately activates transcription of stress response genes (Walsh et al., 2003; De Regt, Baker & Sauer, 2015). DegS is required for the growth of *E. coli*. When *degS* was depleted in *E. coli* MC1061 strain, the cells failed to grow on M9 complete minimal media at 30 and 37 °C (Alba et al., 2001). Furthermore, DegS plays an important role in the virulence of extraintestinal *E. coli* infections, including sepsis, meningitis and urinary tract infections (Redford, Roesch & Welch, 2003). There is growing concern regarding the roles of DegS in *E. coli*, but our knowledge of the roles of this protein in *V. cholerae* remains limited. In *V. cholerae*, OmpU interact with the PDZ-domain of DegS and further activate σ^E^ signaling pathway to resist antimicrobial peptides (Mathur, Davis & Waldor, 2007). Additionally, recent studies have shown that ToxR, a virulence regulator, is also a substrate for *degS* (Lembke et al., 2018). In the present study, we showed that the *degS* deletion mutant significantly impaired the growth of non-O1/non-O139 *V. cholerae*. The *degS* deletion of *V. cholerae* exhibited smaller colonies and slower growth than the wild-type strain. However, the molecular mechanisms remain unknown. To uncover the potential roles of *degS* in *V. cholerae* growth and further investigate the global regulatory role of *degS* in *V. cholerae*, a comparative transcription analysis was performed in the non-O1/non-O139 *V. cholerae* wild-type and *degS* deletion mutant strains using high-throughput RNA sequencing (RNA-Seq) to identify the differentially expressed genes. Gene ontology (GO), pathway analysis and Gene-Act-network analysis were performed to explore the main functions of the differentially expressed genes. We aimed to identify genes that may be involved in *degS* regulation for bacterial growth and guide future studies on the function and regulatory network of *degS*.

## Materials and Methods

### Strain cultivation and DNA manipulation

The *V. cholerae* HN375 strain is a non-O1/non-O139 strain collected by the China Center for Type Culture Collection with accession number CCTCC AB209168 (Luo et al., 2011). This strain was used as the wild-type strain in this study. *V. cholerae* was cultured in Luria Bertani (LB) liquid medium with shaking or on LB agar plates at 37 °C. The *degS* in-frame deletion mutants were generated by the suicide vector pWM91 as described previously (Metcalf et al., 1996). To complement the expression of *degS* and *rpoS* in *degS* mutant strains, the *degS* gene and *rpoS* gene fragments were amplified and then ligated into the expression vector pBAD24 (Guzman et al., 1995). The pBAD24-*degS* and pBAD24-*rpoS* plasmid were transformed into the *V. cholerae degS* mutant, respectively. Subsequently, these strains were cultured in LB medium supplemented with 0.1% arabinose to induce gene expression.

### Bacterial growth experiments

The *V. cholerae* HN375 wild-type and *degS* deletion mutant strains were cultured on Columbia blood agar plates for 16 h at 37 °C. For the plate-based growth experiment, bacteria were picked and inoculated onto blood agar, thiosulfate citrate bile salts sucrose (TCBS) agar, MacConkey agar and LB agar plates using the sectional streak method, and the bacterial morphology was observed after 24 h. The complement strains cultured in LB medium supplemented with 0.1% arabinose, the plate-based growth experiment was performed as described above. Colony size was measured by image J software. The significant differences in the colony diameters were performed with independent-sample *t*-tests using GraphPad Prism 5.0 software (GraphPad Software, San Diego, CA, USA). For the growth curve experiment, a 0.5 McFarland’s standard of fresh bacterial suspension was prepared. The 100-μl bacterial suspensions were seeded into 100 ml of fresh LB liquid medium with shaking at 37 °C, and the OD_600_ values were recorded once per hour. Three technical replicates were performed for each strain. The above experiments were repeated independently three times. The significant differences in the growth curve assays were determined with two-way ANOVA using GraphPad Prism 5.0 software (GraphPad Software, San Diego, CA, USA).

### RNA-Seq data analysis

For in vitro RNA preparation, the wild-type and *degS* deletion mutant strains were grown to mid-exponential phase (OD_600_ of ~0.6) in LB liquid medium. Total RNA was extracted from the wild-type and *degS* deletion mutant using TRIzol reagent (Takara, Dalian, China). After quality control, the cDNA libraries were prepared, and sequencing was performed by NovelBio Bio-Pharm Technology Co. Ltd. (Shanghai, China). Raw reads were generated and filtered. The clean reads were used for mapping to the *V. cholerae* N16961 reference genome (RefSeq accession numbers NC_002505 and NC_002506) using Misplacing software (Wang et al., 2010). Gene expression was determined and normalized using the reads per kilobase per million (Mortazavi et al., 2008). Differential expression between the *degS* deletion mutant and wild-type strains was identified by the DEseq package (Audic & Claverie, 1997). After statistical analysis, the differentially expressed genes were defined according to fold change >1.5 or fold change <0.667 and the false discovery rate (FDR) threshold (FDR < 0.05).

### Gene ontology and pathway analyses

To analyze the main functions of the differentially expressed genes, the differentially expressed genes corresponding to GO terms in the GO database were mapped using the GO-Term Finder tool (Boyle et al., 2004). The differentially expressed genes were annotated in the following three main categories based on the database analysis: biological processes, molecular functions and cellular components. Significantly enriched GO terms were limited to categories with *P*-value < 0.05.

To identify significantly enriched pathways, pathway annotations of the differentially expressed genes were performed according to the Kyoto Encyclopedia of Genes and Genomes (KEGG) database (Aoki-Kinoshita & Kanehisa, 2007). The significantly enriched pathways were selected based on Fisher’s exact test and were defined as having *P*-value < 0.05.

### Gene-Act network

The Gene-Act network was established according to the gene–gene relationships in the KEGG database, including activation, binding, expression, inhibition and compound (Binder & Schumacher, 2008; Wang et al., 2013), which helped identify the main signaling pathways and key regulatory genes.

### Validation of RNA-Seq data by qRT-PCR

To validate the RNA-Seq data, Quantitative real-time PCR (qRT-PCR) was performed to determine the mRNA expression level. Briefly, total RNA from the wild-type and *degS* deletion mutant strains was used for cDNA synthesis using the PrimeScript RT Reagent Kit (Takara, Dalian, China). The qRT-PCR assays were performed using the CFX Connect Real-Time System (Bio-Rad, Hercules, CA, USA) and SYBR Premix Ex Taq Kit (Takara, Dalian, China). The 16s RNA was used as an endogenous reference gene, and the relative changes in gene expression were calculated based on the 2^−ΔΔCt^ method. The primer sequences used are shown in Table S1. Comparisons among groups were performed with independent-sample *t*-tests using GraphPad Prism 5.0 software (GraphPad Software, San Diego, CA, USA).

## Results

### The *degS* gene is important for normal growth of *V. cholerae*

**Figure 1:**
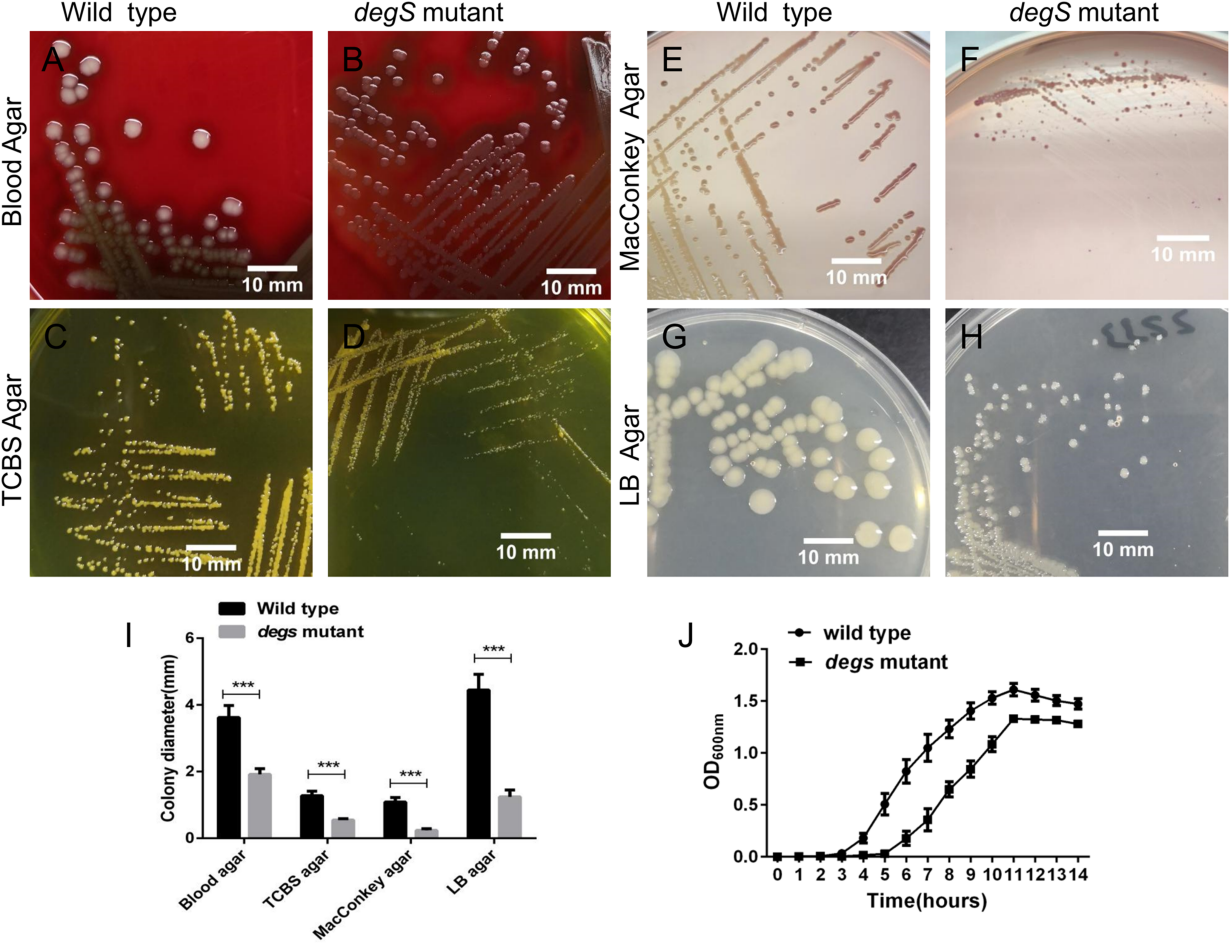
The *degS* deletion affects the colony size and growth curves of *V. cholerae*. (A–H) The small colony phenotype of the *degS* mutant on blood agar, TCBS agar, MacConkey agar and LB agar plates after 24 h of cultivation. (I) Colony diameters. Shown is the average colony diameter of the *degS* mutant and wild-type strains. Error bars represent the standard deviation (SD) of the mean of 10 colonies. ***P < 0.001. (J) Growth curves of the *degS* mutant and wild-type strains. The bacterial densities in LB liquid medium were determined by measuring the absorbance at the indicated time points. The values represent the mean with SD of three independent experiments.

To explore the biological functions of *degS* in *V. cholerae*, we constructed a deletion mutant of *degS* in the *V. cholerae* HN375 strain. The colonies of the *degS* mutant on blood agar, TCBS agar, MacConkey agar and LB agar plates were consistently smaller than those of the wild-type strain (Figs. 1A–1I). Next, we compared the bacterial growth curves of the *degS* mutant and wild-type strains. The *degS* mutant has a longer log phase than the wild-type strain (Fig. 1J). After entering the exponential growth phase, the wild-type and *degS* mutant have similar growth rate, while in the stationary phase, the density of *degS* mutant was lower than that of the wild-type strain. Together, these results suggest that *degS* is important for normal growth of *V. cholerae*.

### Analysis of differential gene expression

**Figure 2:**
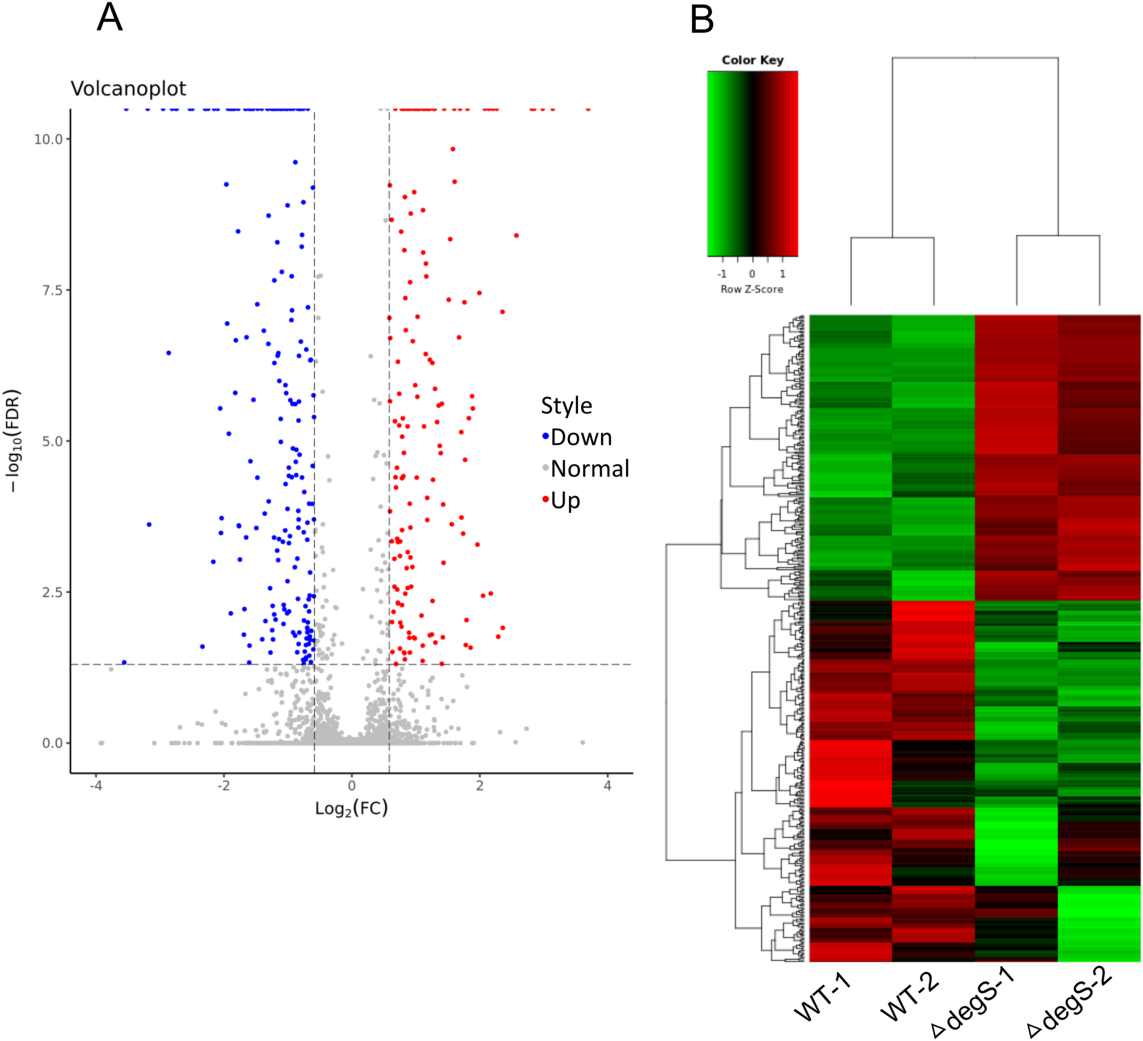
Differentially expressed genes between the *degS* mutant and wild-type (WT) strains. (A) A volcano plot showing the differentially expressed genes. Red represents the upregulated genes, green represents the downregulated genes and gray represents the unchanged genes. (B) A clustering heatmap showing the differentially expressed genes.

To explore the molecular changes in the response to the *degS* deletion mutant in *V. cholerae*, we performed a comparative gene expression analysis between the *degS* deletion mutant and wild-type strains using RNA-Seq. After sequencing and data analysis, the differentially expressed genes were identified using the following criteria: fold change in expression >1.5 or <0.667 and FDR < 0.05. In total, 423 differentially expressed genes were identified (Fig. 2A), including 187 upregulated genes and 236 downregulated genes (Fig. 2B; Table S2). To our knowledge, some of the genes associated with energy metabolism and cell division were also found in this analysis, such as VC0485 (pyruvate kinase), VC2738 (phosphoenolpyruvate carboxykinase), VCA0843 (glyceraldehyde-3-phosphate dehydrogenase) and murB, which may affect bacterial growth. Together, our results show distinct gene profiles between the wild-type and *degS* mutant strains, suggesting that the *degS* deletion mutant can produce unique gene profiles that may be associated with *V. cholerae* growth.

### GO analysis of differentially expressed genes

**Figure 3:**
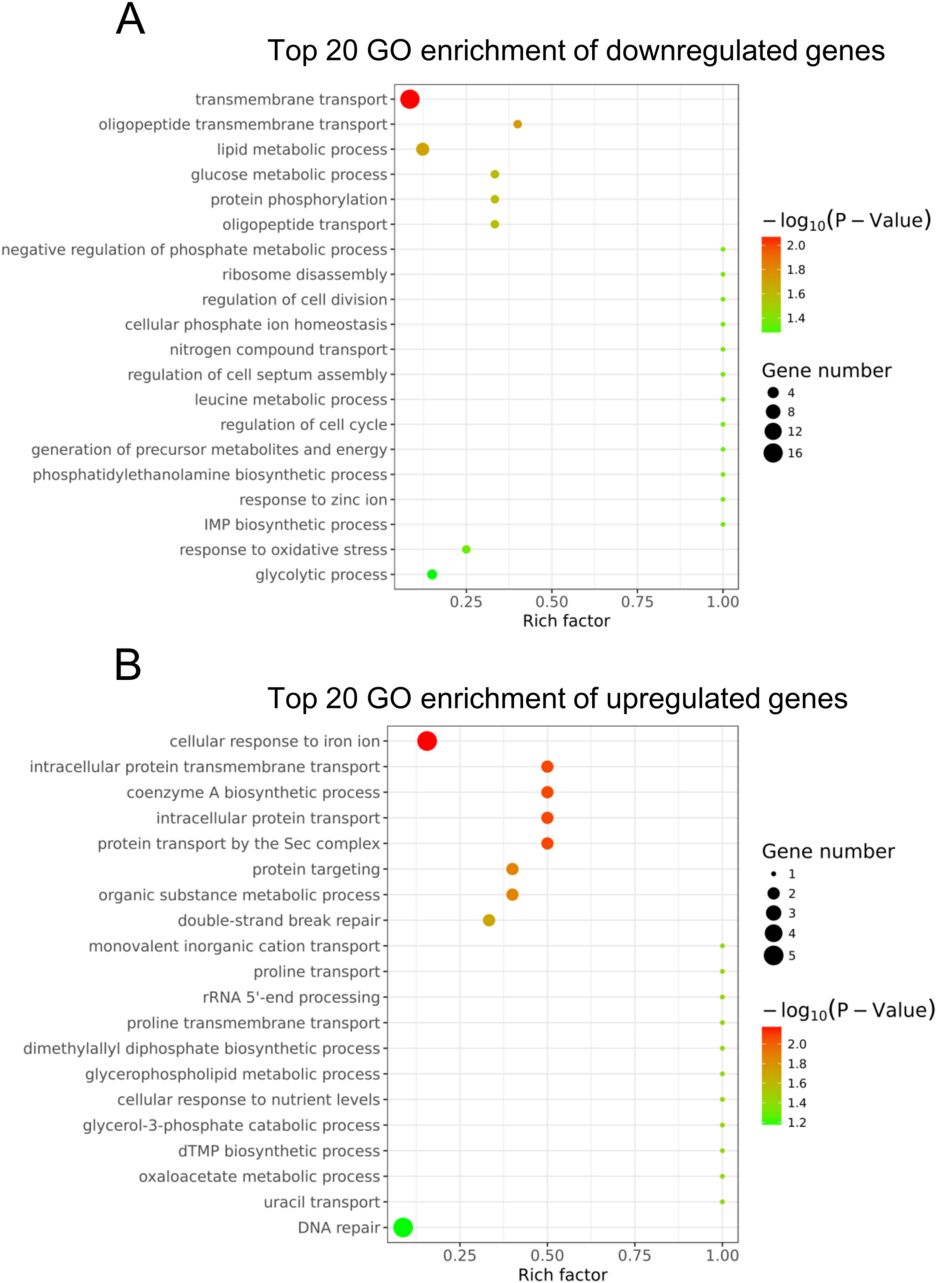
Gene ontology analysis of the differentially expressed genes. (A) The top 20 GO terms annotated in the biological process category for the downregulated genes. (B) The top 20 GO terms annotated in the biological process category for the upregulated genes.

To understand the main functions of the differentially expressed genes, GO analysis was performed according to the GO project. Based on our RNA-Seq data, the primary GO categories for the downregulated GO terms were focused on transmembrane transport, oligopeptide transmembrane transport, lipid metabolic process, glucose metabolic process, negative regulation of phosphate metabolic process, regulation of cell division and others (Fig. 3A). Notably, the GO categories include the lipid metabolic process, glucose metabolic process, negative regulation of the phosphate metabolic process, leucine metabolic process and generation of precursor metabolites and energy, which are related to cellular metabolism. In addition, we found that the GO term categories for the regulation of cell division, regulation of cell septum assembly and regulation of the cell cycle were associated with bacterial growth. The main GO terms for the upregulated genes were the cellular response to iron ion, intracellular protein transmembrane transport, coenzyme A biosynthetic process and others (Fig. 3B).

### Pathway analysis

**Figure 4:**
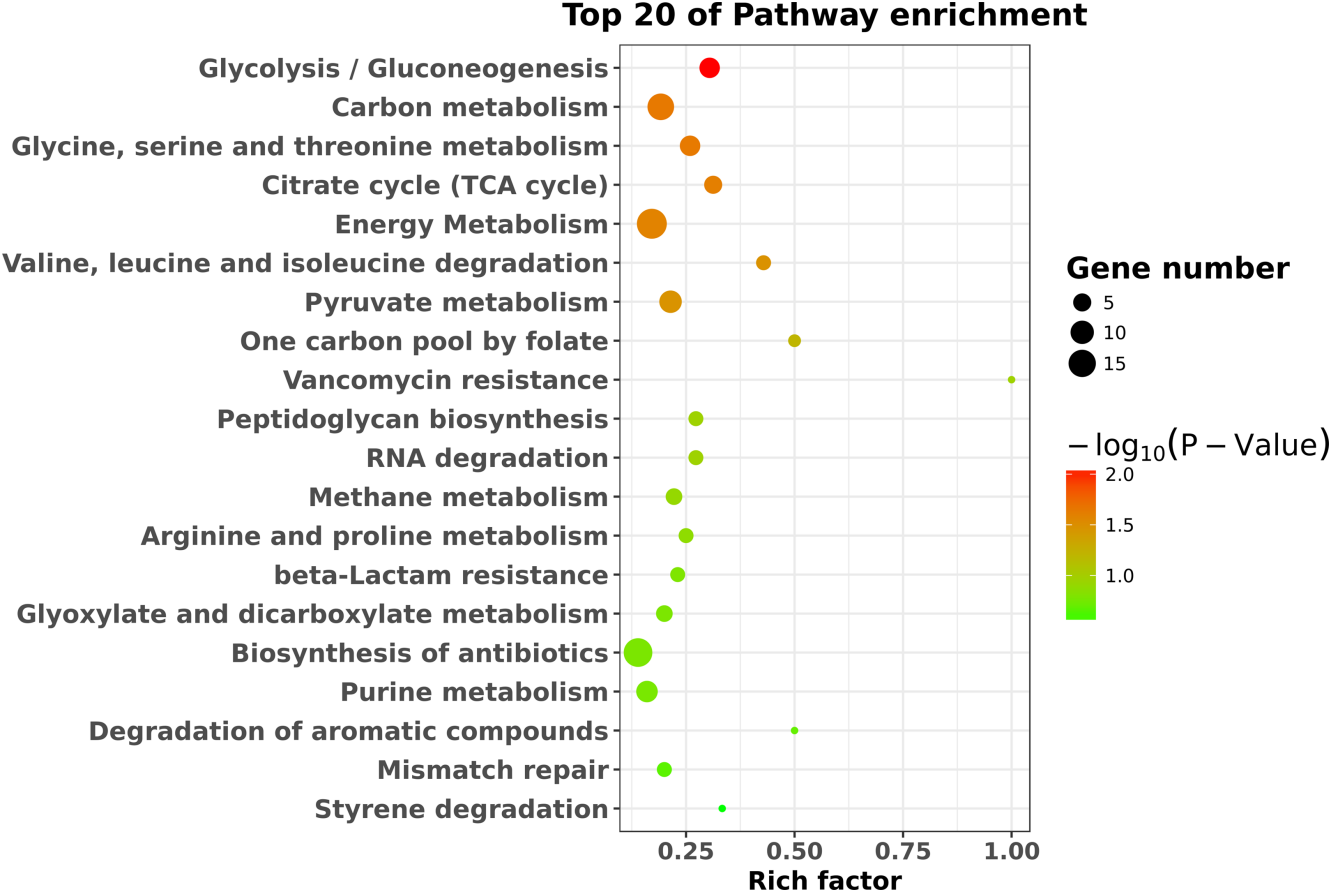
KEGG pathway enrichment analysis of the differentially expressed genes. The top 20 enriched pathways are shown as circles of different sizes and different colors.

To identify the significant pathways, KEGG enrichment pathway analysis was performed based on the KEGG database. The results showed that the 423 differentially expressed genes were mapped to 65 pathways. The top 20 enriched pathways are shown in Fig. 4. Overall, seven statistically significant enriched pathways were defined by the hypergeometric test (Table 1). The statistically significant enriched pathways were mainly involved in glycolysis/gluconeogenesis; carbon metabolism; glycine, serine and threonine metabolism; the citrate cycle (TCA cycle); energy metabolism; valine, leucine and isoleucine degradation; and pyruvate metabolism. These results suggest that there is a regulatory relationship between *degS* and cellular metabolic pathways.

**Table 1:** The significantly enrichment pathways of differentially expressed genes.

Pathway ID	Pathway term	Pathway gene	*P*-value
PATH:00010	Glycolysis/Gluconeogenesis	VC1819, VC2000, VC2738,VC2013, VCA0843, VC0485, VC2638	0.0089266
PATH:01200	Carbon metabolism	VC2504, VC2000, VC1942, VC2738, VC1304, VC2090, VCA0843, VCA0666, VC0485, VC0432, VC2638, VCA0278, VC2646, VC0027	0.0221534
PATH:00260	Glycine, serine and threonine metabolism	VC2504, VC2036, VCA0875, VCA0666, VC2638, VCA0278, VC0027	0.0222798
PATH:00020	Citrate cycle (TCA cycle)	VC2738, VC1304, VC2090, VC0432, VC2638	0.0240401
PATH:01120	Energy metabolism	VC2504, VC1819, VC2000, VC2036, VC1942, VC2738, VC0715, VC0384, VC1304, VC2090, VCA0843, VCA0549, VC1347, VC0485, VC0432, VC2638, VCA0278, VC2646, VC2669	0.0257444
PATH:00280	Valine, leucine and isoleucine degradation	VCA0007, VC2638, VCA0829	0.0328662
PATH:00620	Pyruvate metabolism	VC1819, VC2738, VC0794, VC1304, VC0485, VC0432, VC2638, VCA0192, VC2646	0.0334527

### Gene-Act network analysis

**Figure 5:**
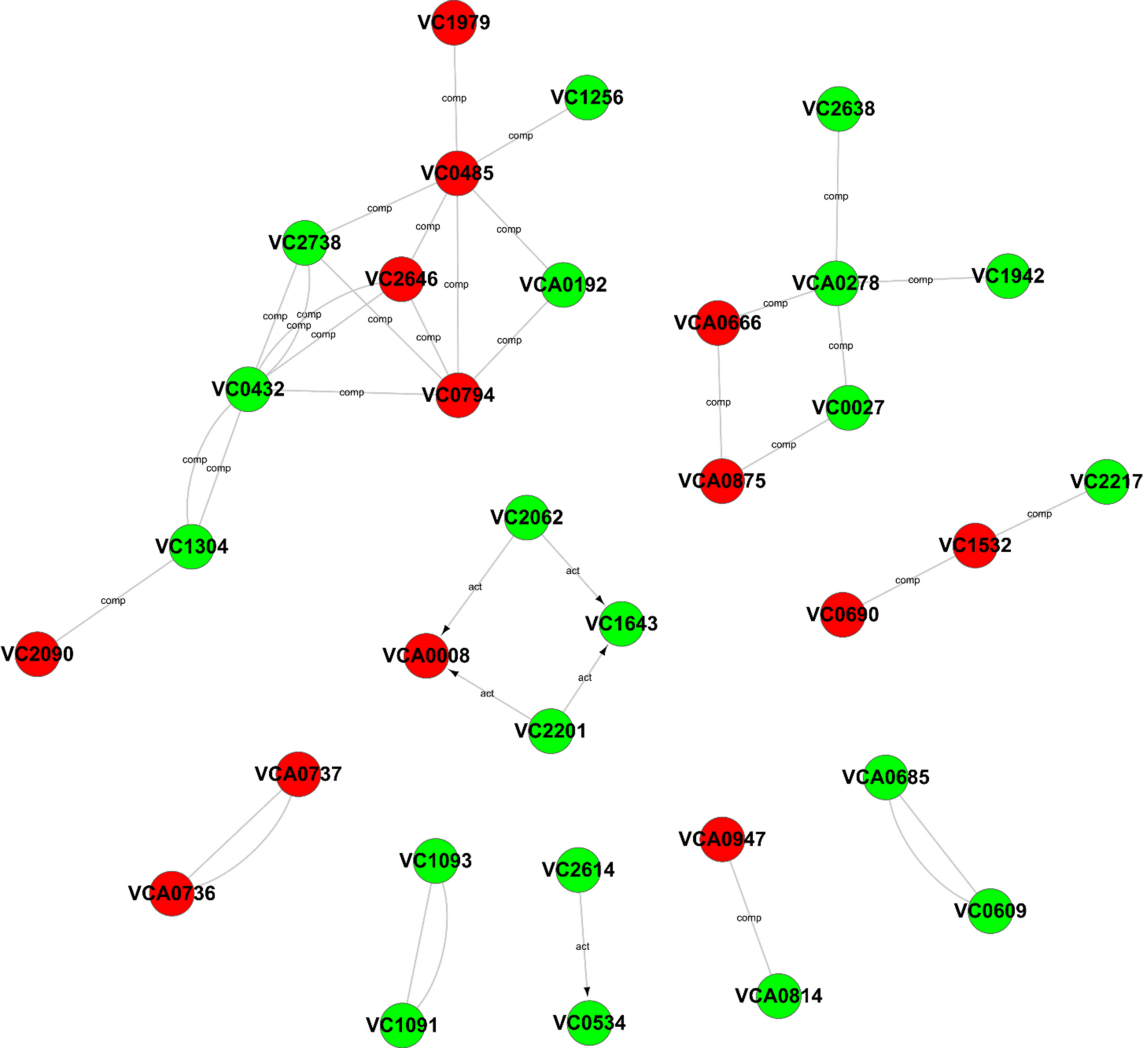
Gene-Act network of differentially expressed genes. The red circle nodes indicate the upregulated genes, and the green circle nodes indicate the downregulated genes. The lines indicate interactions between genes.

To further explore the relationships among these differentially expressed genes, a Gene-Act network was constructed based on the genes and genetic relationships, including activation, binding, expression, inhibition and compounds. According to the Gene-Act network shown in Fig. 5, important network modules, including VC2738 (phosphoenolpyruvate carboxykinase), VC2646 (phosphoenolpyruvate carboxylase), VC0485 (pyruvate kinase), VC0432 (malate dehydrogenase), VC1304 (fumarate hydratase, class I), VC2090 (the succinate dehydrogenase hydrophobic membrane anchor protein), VC0794 (hypothetical protein) and VCA0192 (D-lactate dehydrogenase), were involved in multiple cellular metabolic pathways, as mentioned in Table 1 above. We also found that VCA0278 (serine hydroxymethyltransferase), VCA0666 (L-serine dehydratase 1), VCA0875 (D-serine dehydratase), VC0027 (threonine dehydratase) and VC2638 (dihydrolipoamide dehydrogenase) were involved in glycine, serine and threonine metabolism. Interestingly, the interaction of two important genes, namely, VCA0737 (the luxP protein) and VCA0736 (the sensor histidine kinase LuxQ), in the quorum sensing system and the interaction of two important genes, namely, VC2614 (the cAMP-regulatory protein) and VC0534 (the RNA polymerase sigma-38 factor, rpoS), in the cAMP-CRP-RpoS signaling pathway, were also observed in the gene interaction network. In addition, four genes, namely, VCA0008 (amethyl-accepting chemotaxis protein), VC2201 (the chemotaxis protein CheR), VC1643 (amethyl-accepting chemotaxis protein) and VC2062 (achemotaxis-specific methylesterase), contribute to the bacterial chemotaxis pathway and were also affected by the *degS* mutant.

### Validation of the RNA-Seq data

**Figure 6:**
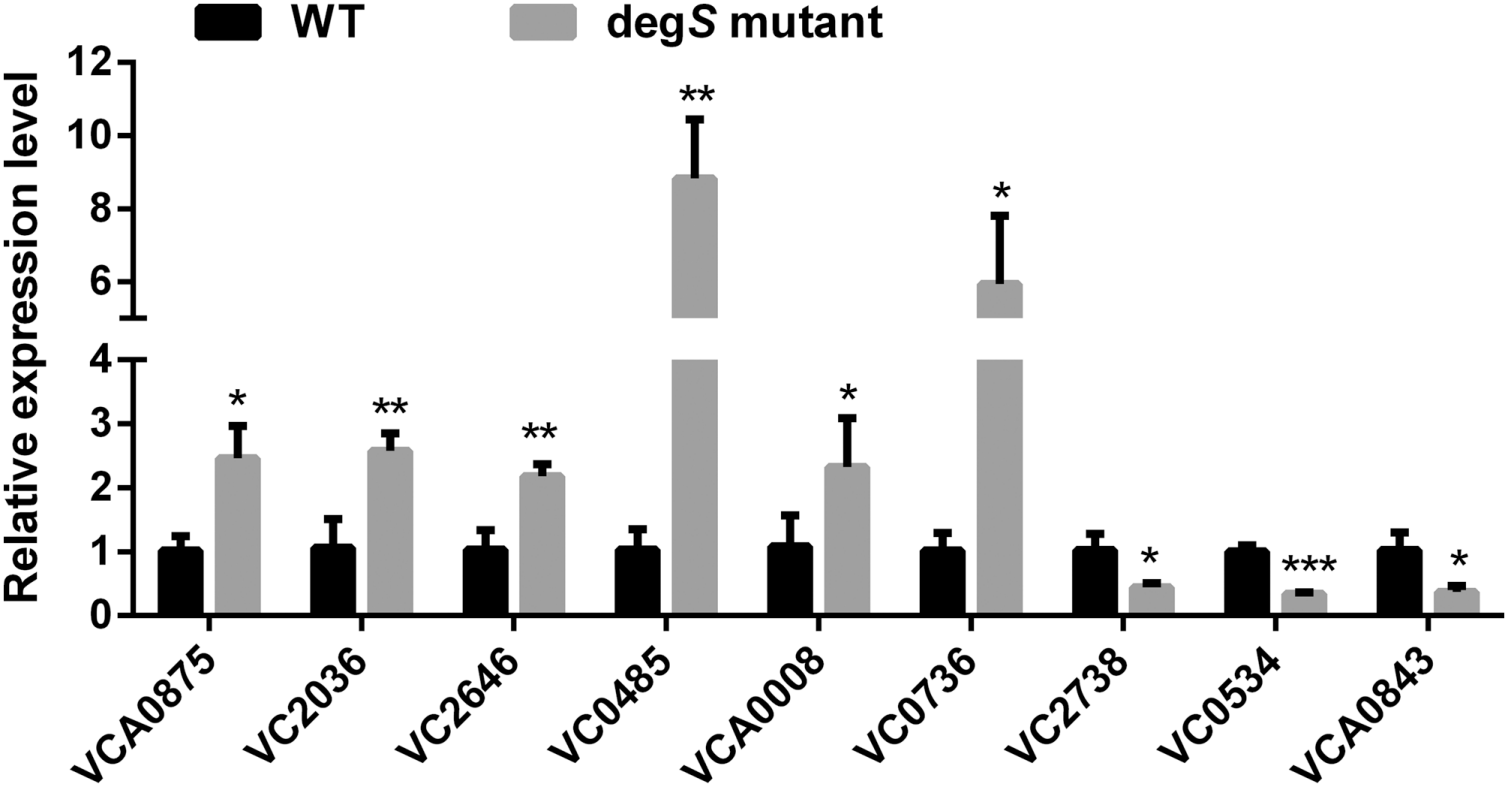
Validation of the differentially expressed genes using quantitative real-time PCR. The relative expression values of genes are presented as the means with SD. *P < 0.05, **P < 0.01 and ***P < 0.001, with comparisons between groups.

To validate the reliability and accuracy of the RNA-Seq analysis, nine differentially expressed genes were randomly selected for validation by qRT-PCR. As shown in Fig. 6, compared with the *V. cholerae* wild-type strain, the differentially expressed genes VCA0875, VC2036, VC2646, VC0485, VCA0008 and VC0736 were significantly upregulated, while VC2738, VC0534 and VCA0843 were significantly downregulated, in the *degS* mutant strain. These results were consistent with those of the RNA-Seq analysis, indicating the reliability and accuracy of the RNA-Seq data.

### The small colony *degS* mutant phenotype is rescued by supplementing the expression of *degS* and *rpoS* gene

**Figure 7:**
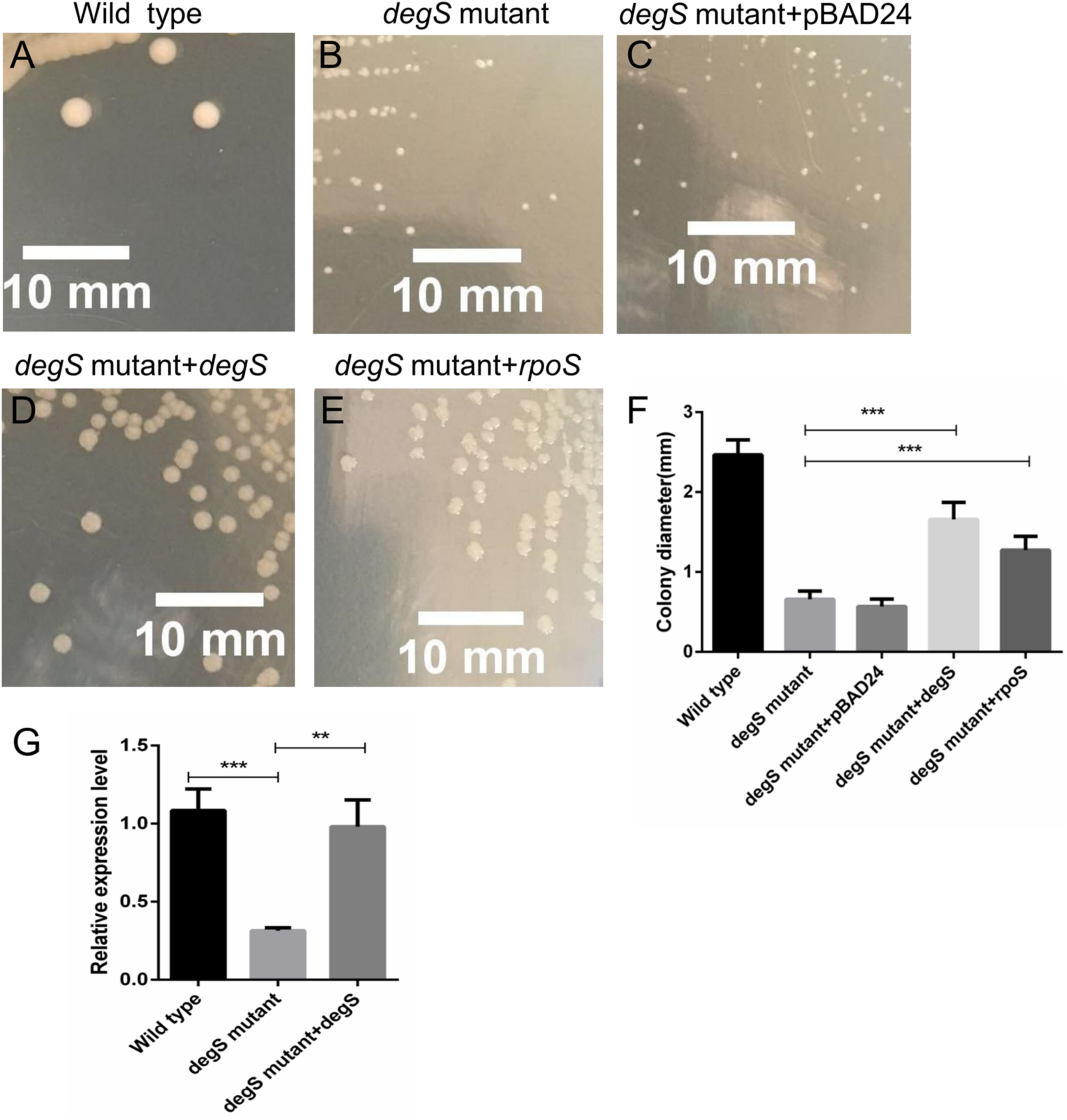
Compensation for *degS* and *rpoS* expression in *degS* deletion mutant could restore the colony size. (A–E) The cell morphology and (F) average colony diameter of the wild-type, *degS* mutant and *degS* mutant complemented with the plasmids pBAD24, pBAD24-*degS* and pBAD24-*rpoS*, respectively. Error bars represent the SD of the mean of 10 colonies. ***P < 0.001. (G) The mRNA expression levels of *rpoS* were analyzed in wild-type, *degS* mutant and *degS* mutant complemented with the pBAD24-*degS* plasmids. The relative expression values of genes are presented as the means with SD. **P < 0.01 and ***P < 0.001.

To further test the role of *degS* in the small colony phenotype, we constructed pBAD24-*degS* plasmid to complement *degS* gene in *degS* mutant strains. The small colony phenotype could be restored in *degS* mutants by complementation with the pBAD24-*degS* plasmid (Figs. 7A–7F). According to the above Gene-Act network, the cAMP-CRP-RpoS signaling pathway was significantly inhibited in the *degS* deletion mutant. We further confirmed that the expression of *rpoS* was inhibited in *degS* mutants and restored in *degS* mutants by complementation with the pBAD24-*degS* plasmid (Fig. 7G). RpoS has been reported to aid bacterial growth and survive in stationary phase (McCann, Kidwell & Matin, 1991; Shah et al., 2019). Therefore, to test whether *rpoS* is involved in the small colony *degS* mutant phenotype, we constructed pBAD24-*rpoS* plasmid to supplement the expression of *ropS* gene in *degS* mutant strains. The results showed that the small colony phenotype could be partially restored by supplement the expression of *ropS* gene.

## Discussion

DegS is a serine protease located in the periplasm of bacteria, and is an important component of the bacterial response to external pressure (Kim, 2015). The *degS* is now seen as an essential gene whose indispensable function is to provide active σ^E^ activity (Alba et al., 2001; Pennetzdorfer et al., 2019). However, the biological function and global regulatory network of DegS in *V. cholerae* remain limited. In this study, we report that *degS* deletion could significantly delay the normal growth of *V. cholerae* in the liquid medium and showed a small colony phenotype on agar plates. The small colony phenotype of *degS* mutant could be reversed by complementation with the pBAD24-*degS* plasmid encoding the full-length DegS protein. The consistent results were reported that *degS* required for growth in three *E. coli* strains (Alba et al., 2001). Interestingly, previous studies have found that an *E. coli degS* knockout strain also showed smaller colonies on LB plates than the wild-type strain, but the molecular mechanism is not clear (Waller & Sauer, 1996). To investigate the global regulatory role of *degS* in *V. cholerae*, we further performed a genome-wide analysis of mRNAs from the *V. cholerae* HN375 wild-type and *degS* mutant strains. To our knowledge, this is the first genome-wide analysis of mRNAs performed to screen for differentially expressed genes induced by the *degS* deletion in *V. cholerae*. Therefore, we believe that these data will serve as a useful resource for further research on the overall regulatory role of *degS*.

The RNA-Seq data presented here show that 423 differentially expressed genes were identified. Among the downregulated enriched GO terms, we found that the murB gene was associated with the GO terms of cell cycle and cell division. The murB gene encodes the UDP-N-acetylenolpyruvoylglucosamine reductase, which catalyzes a critical process in the peptidoglycan biosynthetic pathway (Kedar et al., 2007). In *Bacillus subtilis*, normal expression of the murB gene plays an important role in the maintenance of normal bacterial growth and morphology (Real & Henriques, 2006). In addition, according to the GO analysis, the downregulated genes VC0263 (galactosyl-transferase), VC2217 (beta-N-acetylhexosaminidase) and VC1956 (lytic murein transglycosylase) were involved in the polysaccharide biosynthetic process, peptidoglycan turnover and peptidoglycan-based cell wall biogenesis, respectively. The synthesis of peptidoglycans is closely associated with bacterial cell division and growth. In this study, deletion of *degS* inhibited the growth of *V. cholerae*, possibly via repression of peptidoglycan synthesis.

In this study, we demonstrated for the first time that *degS* is closely associated with cellular metabolism in the model bacterium *V. cholerae*. According to GO and KEGG pathway analyses, we showed that deletion of *degS* mainly repressed glycolysis/gluconeogenesis, the citrate cycle (TCA cycle), carbon metabolism and pyruvate metabolism. Cellular metabolism is a well-known key player in the nutrient regulation of bacterial division and the cell cycle. The growth of bacteria in the natural environment is strongly influenced by changes in nutrient availability (Schaechter, MaalØe & Kjeldgaard, 1958; Woldringh et al., 1980). Many metabolites and metabolic enzymes have been found to play a direct regulatory role in the critical processes of bacterial growth and division (Weart et al., 2007). Pyruvate, as the final metabolite of glycolysis, plays an important role in the coordination of bacterial growth and division (Monahan et al., 2014). Several enzymes implicated in central carbon metabolism, such as the E1α subunit of pyruvate dehydrogenase, phosphoglyceromutase and enolase, are involved in coordination of the cell cycle and cell growth (Hill et al., 2013; Vadia & Levin, 2015; Sperber & Herman, 2017). Our findings indicate that *degS* may affect bacterial growth by regulating bacterial metabolism.

In addition, according to the Gene-Act network analysis, the cAMP-CRP-RpoS signaling pathway was significantly inhibited in the *degS* deletion mutant. cAMP-CRP and the alternative sigma factor RpoS are two global transcriptional regulatorsin bacteria. The cAMP-CRP complex can bind to the promoter region of *rpoS* and further stimulate the transcription of this gene (Cheng & Sun, 2009). RpoS is an alternative sigma factor (σ^S^) of RNA polymerase that plays a role in the general stress response and modulation of the stationary phase (Weber et al., 2005; Pennetzdorfer et al., 2019). In *V. cholerae*, *rpoS* mutants are highly sensitive to hydrogen peroxide and are involved in colonization during the infection process and in the dissolution of biofilms (Merrell et al., 2000; Müller et al., 2007). It was shown that RpoS is necessary for upregulation of the chemotaxis and motility genes of *V. cholerae* within the rabbit intestine (Nielsen et al., 2006; Wurm et al., 2017). In our transcriptomic data, some flagellum- and chemotaxis-related genes that regulated by RpoS (Table S2; Fig. 5), such as flhF (flagellar biosynthesis regulator FlhF), VC2141 (flagellar protein FlaG), VC2201 (chemotaxis protein CheR), VC1643 (amethyl-accepting chemotaxis protein) (Nielsen et al., 2006), were significantly inhibited in the *degS* deletion mutant. Previous studies have found that an *E. coli rpoS* mutant was less able to survive in stationary phase (McCann, Kidwell & Matin, 1991). Additionally, *rpoS* may aid the growth of *Salmonella Newport* strain in amended soil extract (Shah et al., 2019). In this study, we observed that the small colony *degS* mutant phenotype could be partially restored in *degS* mutant with pBAD24-*rpoS* plasmid. These data suggest that the cAMP-CRP-RpoS signaling pathway may be involved in the small colony *degS* mutant phenotype. Furthermore, the qRT-PCR results showed that the expression of *rpoS* gene was positively correlated with the expression of *degS*, suggesting that *degS* may be associated with the cAMP-CRP-RpoS signaling pathway, and the specific mechanism needs further investigation. In addition, the LuxPQ signal transduction system is a quorum sensing system restricted to Vibrionales (Brackman et al., 2009). The LuxPQ signal system was significantly activated after *degS* deletion, suggesting that LuxP and LuxQ may be *degS*-repressed genes.

## Conclusions

This study shows that DegS is important for normal growth of *V. cholerae*. When *degS* was deleted, *V. cholerae* exhibited smaller colonies on various media and slower growth than the wild-type strain. RNA-Seq analysis results revealed that some of the differentially expressed genes were involved in various cellular metabolic processes and the cell cycle, which may be associated with bacterial growth. In addition, several new *degS*-related regulatory networks have been identified. The cAMP-CRP-RpoS signaling pathway may be involved in the small colony *degS* mutant phenotype. Together, the current results not only provide important information for future research but also extend our understanding of the regulatory network of *degS*.

## Supplemental Information

10.7717/peerj.7959/supp-1 Supplemental Information 1 Primers for quantitative real-time PCR.

10.7717/peerj.7959/supp-2 Supplemental Information 2 Differentially expressed genes between the degS deletion mutant and wild type strain.

## Additional Information and Declarations

### Data Availability

The following information was supplied regarding data availability: The raw data is available at NCBI GEO: GSE132870.

## References

[ref-1] Alba BM, Zhong HJ, Pelayo JC, Gross CA (2001). *degS* (*hhoB*) is an essential *Escherichia coli* gene whose indispensable function is to provide σ^E^ activity. Molecular Microbiology.

[ref-2] Ali M, Lopez AL, You YA, Kim YE, Sah B, Maskery B, Clemens J (2012). The global burden of cholera. Bulletin of the World Health Organization.

[ref-3] Aoki-Kinoshita KF, Kanehisa M (2007). Gene annotation and pathway mapping in KEGG. Methods in Molecular Biology.

[ref-4] Audic S, Claverie JM (1997). The significance of digital gene expression profiles. Genome Research.

[ref-5] Aydanian A, Tang L, Chen Y, Morris JG, Olsen P, Johnson JA, Nair GB, Stine OC (2015). Genetic relatedness of selected clinical and environmental non-O1/O139 Vibrio cholerae. International Journal of Infectious Diseases.

[ref-6] Binder H, Schumacher M (2008). Comment on ‘network-constrained regularization and variable selection for analysis of genomic data’. Bioinformatics.

[ref-7] Boyle EI, Weng S, Gollub J, Jin H, Botstein D, Cherry JM, Sherlock G (2004). GO::TermFinder--open source software for accessing Gene Ontology information and finding significantly enriched Gene Ontology terms associated with a list of genes. Bioinformatics.

[ref-8] Brackman G, Celen S, Baruah K, Bossier P, Van Calenbergh S, Nelis HJ, Coenye T (2009). AI-2 quorum-sensing inhibitors affect the starvation response and reduce virulence in several *Vibrio* species, most likely by interfering with LuxPQ. Microbiology.

[ref-9] Chatterjee SN, Chaudhuri K (2003). Lipopolysaccharides of *Vibrio cholerae*. I. Physical and chemical characterization. Biochimica et Biophysica Acta.

[ref-10] Cheng Y, Sun B (2009). Polyphosphate kinase affects oxidative stress response by modulating cAMP receptor protein and rpoS expression in *Salmonella typhimurium*. Journal of Microbiology and Biotechnology.

[ref-11] De Regt AK, Baker TA, Sauer RT (2015). Steric clashes with bound OMP peptides activate the DegS stress-response protease. Proceedings of the National Academy of Sciences of the United States of America.

[ref-12] Guzman LM, Belin D, Carson MJ, Beckwith J (1995). Tight regulation, modulation, and high-level expression by vectors containing the arabinose PBAD promoter. Journal of Bacteriology.

[ref-13] Hao Y, Wang Y, Bi Z, Sun B, Jin Y, Bai Y, Chen B, Shao C, Sun X, Lu Z (2015). A case of non-O1/non-O139 *Vibrio cholerae* septicemia and meningitis in a neonate. International Journal of Infectious Diseases.

[ref-14] Hill NS, Buske PJ, Shi Y, Levin PA (2013). A moonlighting enzyme links *Escherichia coli* cell size with central metabolism. PLOS Genetics.

[ref-15] Kedar GC, Brown-Driver V, Reyes DR, Hilgers MT, Stidham MA, Shaw KJ, Finn J, Haselbeck RJ (2007). Evaluation of the metS and murB loci for antibiotic discovery using targeted antisense RNA expression analysis in *Bacillus anthracis*. Antimicrobial Agents and Chemotherapy.

[ref-16] Kim DY (2015). Two stress sensor proteins for the expression of sigmaE regulon: DegS and RseB. Journal of Microbiology.

[ref-17] Lembke M, Pennetzdorfer N, Tutz S, Koller M, Vorkapic D, Zhu J, Schild S, Reidl J (2018). Proteolysis of ToxR is controlled by cysteine-thiol redox state and bile salts in *Vibrio cholerae*. Molecular Microbiology.

[ref-18] Luo P, Su T, Hu C, Ren C (2011). A novel and simple PCR walking method for rapid acquisition of long DNA sequence flanking a known site in microbial genome. Molecular Biotechnology.

[ref-19] Mathur J, Davis BM, Waldor MK (2007). Antimicrobial peptides activate the *Vibrio cholerae* σ^E^ regulon through an OmpU-dependent signalling pathway. Molecular Microbiology.

[ref-20] McCann MP, Kidwell JP, Matin A (1991). The putative sigma factor KatF has a central role in development of starvation-mediated general resistance in *Escherichia coli*. Journal of Bacteriology.

[ref-21] Merrell DS, Tischler AD, Lee SH, Camilli A (2000). *Vibrio cholerae* requires rpoS for efficient intestinal colonization. Infection and Immunity.

[ref-22] Metcalf WW, Jiang W, Daniels LL, Kim SK, Haldimann A, Wanner BL (1996). Conditionally replicative and conjugative plasmids carrying *lacZ*α for cloning, mutagenesis, and allele replacement in bacteria. Plasmid.

[ref-23] Monahan LG, Hajduk IV, Blaber SP, Charles IG, Harry EJ, Gottesman S (2014). Coordinating bacterial cell division with nutrient availability: a role for glycolysis. mBio.

[ref-24] Mortazavi A, Williams BA, McCue K, Schaeffer L, Wold B (2008). Mapping and quantifying mammalian transcriptomes by RNA-Seq. Nature Methods.

[ref-25] Müller J, Miller MC, Nielsen AT, Schoolnik GK, Spormann AM (2007). *vpsA*-and *luxO*-independent biofilms of *Vibrio cholerae*. FEMS Microbiology Letters.

[ref-26] Mutreja A, Kim DW, Thomson NR, Connor TR, Lee JH, Kariuki S, Croucher NJ, Choi SY, Harris SR, Lebens M, Niyogi SK, Kim EJ, Ramamurthy T, Chun J, Wood JLN, Clemens JD, Czerkinsky C, Nair GB, Holmgren J, Parkhill J, Dougan G (2011). Evidence for several waves of global transmission in the seventh cholera pandemic. Nature.

[ref-27] Nielsen AT, Dolganov NA, Otto G, Miller MC, Wu CY, Schoolnik GK (2006). RpoS controls the *Vibrio cholerae* mucosal escape response. PLOS Pathogens.

[ref-28] Pennetzdorfer N, Lembke M, Pressler K, Matson JS, Reidl J, Schild S (2019). Regulated proteolysis in *Vibrio cholerae* allowing rapid adaptation to stress conditions. Frontiers in Cellular and Infection Microbiology.

[ref-29] Petsaris O, Nousbaum JB, Quilici ML, Le Coadou G, Payan C, Abalain ML (2010). Non-O1, non-O139 *Vibrio cholerae* bacteraemia in a cirrhotic patient. Journal of Medical Microbiology.

[ref-30] Real G, Henriques AO (2006). Localization of the *Bacillus subtilis* murB gene within the dcw cluster is important for growth and sporulation. Journal of Bacteriology.

[ref-31] Redford P, Roesch PL, Welch RA (2003). *degS* is necessary for virulence and is among extraintestinal *Escherichia coli* genes induced in murine peritonitis. Infection and Immunity.

[ref-32] Schaechter M, MaalØe O, Kjeldgaard NO (1958). Dependency on medium and temperature of cell size and chemical composition during balanced grown of *Salmonella typhimurium*. Journal of General Microbiology.

[ref-33] Shah MK, Bradshaw R, Nyarko E, Millner PD, Neher D, Weicht T, Bergholz TM, Sharma M (2019). Survival and growth of wild-type and *rpoS*-deficient *Salmonella* newport strains in soil extracts prepared with heat-treated poultry pellets. Journal of Food Protection.

[ref-34] Sperber AM, Herman JK (2017). Metabolism shapes the cell. Journal of Bacteriology.

[ref-35] Trubiano JA, Lee JYH, Valcanis M, Gregory J, Sutton BA, Holmes NE (2014). Non-O1, non-O139 *Vibrio cholerae* bacteraemia in an Australian population. Internal Medicine Journal.

[ref-36] Vadia S, Levin PA (2015). Growth rate and cell size: a re-examination of the growth law. Current Opinion in Microbiology.

[ref-37] Waller PR, Sauer RT (1996). Characterization of degQ and degS, *Escherichia coli* genes encoding homologs of the DegP protease. Journal of Bacteriology.

[ref-38] Walsh NP, Alba BM, Bose B, Gross CA, Sauer RT (2003). OMP peptide signals initiate the envelope-stress response by activating DegS protease via relief of inhibition mediated by its PDZ domain. Cell.

[ref-39] Wang K, Singh D, Zeng Z, Coleman SJ, Huang Y, Savich GL, He X, Mieczkowski P, Grimm SA, Perou CM, MacLeod JN, Chiang DY, Prins JF, Liu J (2010). MapSplice: accurate mapping of RNA-seq reads for splice junction discovery. Nucleic Acids Research.

[ref-40] Wang M, Verdier J, Benedito VA, Tang Y, Murray JD, Ge Y, Becker JD, Carvalho H, Rogers C, Udvardi M, He J (2013). LegumeGRN: a gene regulatory network prediction server for functional and comparative studies. PLOS ONE.

[ref-41] Weart RB, Lee AH, Chien AC, Haeusser DP, Hill NS, Levin PA (2007). A metabolic sensor governing cell size in bacteria. Cell.

[ref-42] Weber H, Polen T, Heuveling J, Wendisch VF, Hengge R (2005). Genome-wide analysis of the general stress response network in *Escherichia coli*: σ^S^-dependent genes, promoters, and sigma factor selectivity. Journal of Bacteriology.

[ref-43] Woldringh CL, Grover NB, Rosenberger RF, Zaritsky A (1980). Dimensional rearrangement of rod-shaped bacteria following nutritional shift-up. II. Experiments with *Escherichia coli* B/r. Journal of Theoretical Biology.

[ref-44] Wurm P, Tutz S, Mutsam B, Vorkapic D, Heyne B, Grabner C, Kleewein K, Halscheidt A, Schild S, Reidl J (2017). Stringent factor and proteolysis control of sigma factor RpoS expression in *Vibrio cholerae*. International Journal of Medical Microbiology.

[ref-45] Zeth K (2004). Structural analysis of DegS, a stress sensor of the bacterial periplasm. FEBS Letters.

