# Supplementary material for: Exploration of the effects of a degS mutant on the growth of Vibrio cholerae and the global regulatory function of degS by RNA sequencing
Source: PeerJ. 2019 Oct 23;7:e7959. doi: 10.7717/peerj.7959 (PMC6815195; doi:10.7717/peerj.7959)
Supplement: Supplemental Information 1 [file peerj-07-7959-s001.doc]

| Primer | Sequence（5'-3'） | product size (bp) |
| --- | --- | --- |
| VCA0875-F | TGCTTGGTGTTCATACTGGCTTGC | 177 |
| VCA0875-R | GCAGTCGGTACATACGCTCATCAC |  |
| VC2036-F | GCGTGGCATGGTCGGTTCTG | 192 |
| VC2036-R | TGGCAGGTAATGACAGCATCAAGC |  |
| VC2646-F | TGATTCGTCAGCAGCGTCCAAC | 84 |
| VC2646-R | GAACGGCGTGCCACAGTGAG |  |
| VC0485-F | TGACTACGTTGAACACGGCACTC | 151 |
| VC0485-R | GCCAGCGACCAGATCCACATC |  |
| VCA0008-F | AAGAGACCAATGCCATGAGCCAAC | 96 |
| VCA0008-R | CGGTCATCGCTGTAGCCACTTG |  |
| VC0736-F | CATGCTGTCGTGGTGGTGATGG | 187 |
| VC0736-R | CGTTCTTGGCTGTGCTGTCCTG |  |
| VC2738-F | GAGCGTGGCATTACTGAACCTACC | 157 |
| VC2738-R | CGCTGCCGTTCCAACCTGTG |  |
| VC0534-F | GTTGCTGTTGAAGGGGCAAG | 183 |
| VC0534-R | AATCATGCGTTTACGTGCGG |  |
| VCA0843-F | CCGCATTAGACGCCGCACTAC | 161 |
| VCA0843-R | TCCACCACGCACAACAATCGC |  |
| rpoS-F | GTTGCTGTTGAAGGGGCAAG | 182 |
| rpoS-R | AATCATGCGTTTACGTGCGG |  |
